# Cerebellar–cortical dysconnectivity in resting‐state associated with sensorimotor tasks in schizophrenia

**DOI:** 10.1002/hbm.25002

**Published:** 2020-04-06

**Authors:** Dae‐Jin Kim, Alexandra B. Moussa‐Tooks, Amanda R. Bolbecker, Deborah Apthorp, Sharlene D. Newman, Brian F. O'Donnell, William P. Hetrick

**Affiliations:** ^1^ Department of Psychological and Brain Sciences Indiana University Bloomington Indiana USA; ^2^ Program in Neuroscience Indiana University Bloomington Indiana USA; ^3^ Department of Psychiatry Indiana University School of Medicine Indianapolis Indiana USA; ^4^ School of Psychology, Faculty of Medicine and Health University of New England Armidale New South Wales Australia; ^5^ Research School of Computer Science, College of Engineering and Computer Science Australian National University Canberra Australian Capital Territory Australia

**Keywords:** cerebellum, functional connectivity, schizophrenia, finger tapping, postural sway

## Abstract

Abnormalities of cerebellar function have been implicated in the pathophysiology of schizophrenia. Since the cerebellum has afferent and efferent projections to diverse brain regions, abnormalities in cerebellar lobules could affect functional connectivity with multiple functional systems in the brain. Prior studies, however, have not examined the relationship of individual cerebellar lobules with motor and nonmotor resting‐state functional networks. We evaluated these relationships using resting‐state fMRI in 30 patients with a schizophrenia‐spectrum disorder and 37 healthy comparison participants. For connectivity analyses, the cerebellum was parcellated into 18 lobular and vermal regions, and functional connectivity of each lobule to 10 major functional networks in the cerebrum was evaluated. The relationship between functional connectivity measures and behavioral performance on sensorimotor tasks (i.e., finger‐tapping and postural sway) was also examined. We found cerebellar–cortical hyperconnectivity in schizophrenia, which was predominantly associated with Crus I, Crus II, lobule IX, and lobule X. Specifically, abnormal cerebellar connectivity was found to the cerebral ventral attention, motor, and auditory networks. This cerebellar–cortical connectivity in the resting‐state was differentially associated with sensorimotor task‐based behavioral measures in schizophrenia and healthy comparison participants—that is, dissociation with motor network and association with nonmotor network in schizophrenia. These findings suggest that functional association between individual cerebellar lobules and the ventral attentional, motor, and auditory networks is particularly affected in schizophrenia. They are also consistent with dysconnectivity models of schizophrenia suggesting cerebellar contributions to a broad range of sensorimotor and cognitive operations.

## Graphical abstract

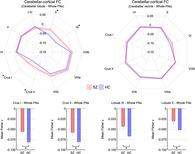

## 1 INTRODUCTION

It has been hypothesized that dysconnectivity between the frontal cortex, thalamus, and cerebellum might produce “cognitive dysmetria” in persons with schizophrenia‐spectrum disorders (Andreasen et al., 1999). When the cognitive dysmetria model was proposed, there was scant research on the cerebellum in schizophrenia. Since then, converging evidence has provided compelling support for cerebellar pathology and dysfunction. Anatomical findings include gray matter deficits (Wolfers et al., 2018), reduced white matter integrity (Mamah, Ji, Rutlin, & Shimony, 2019), disrupted connectivity (Klauser et al., 2017) and modularity (D. J. Kim et al., 2014) in the cerebellum in schizophrenia. Such structural abnormalities contribute to impairments in cognitive processes (Andreasen et al., 1996; Andreasen & Pierson, 2008; Ridler et al., 2006) and contribute to sensorimotor dysfunction—for example, postural sway (Apthorp, Bolbecker, Bartolomeo, O'Donnell, & Hetrick, 2019), sensorimotor synchronization (Moussa‐Tooks et al., 2019), and prediction (Shergill et al., 2014) mediated by the cerebellum. Resting‐state functional connectivity disturbances have also been detected. Specifically, functional dysconnectivity within the thalamo‐cerebellar, cortico‐cerebellar, and sensorimotor networks in resting‐state have been observed in schizophrenia (Anticevic et al., 2014; Berman et al., 2016; Kaufmann et al., 2015; Walther et al., 2017). However, few studies of resting‐state functional connectivity have applied anatomical parcellation to identify specific cerebellar lobules or regions affected in schizophrenia. In a seminal study, Buckner et al. utilized a functional parcellation of the cerebellum based on the cerebral functional networks (Buckner, Krienen, Castellanos, Diaz, & Yeo, 2011; Yeo et al., 2011). These functional parcellations for cerebellar resting‐state connectivity, however, assigned cerebellar regions only to their most dominant principal cerebral targets using a winner‐take‐all algorithm, which might miss secondary functional associations from cerebellar seed regions (Yeo et al., 2011).

While the cerebellum constitutes only 10% of total brain mass, it has about four times as many neurons as the cerebral cortex, representing 80% of total brain neurons (Azevedo et al., 2009). The cerebellum is heterogeneous in terms of its structure and function. Anatomically, it consists of four different types of neurons in the cerebellar cortex—that is, granule cells, Purkinje cells, Golgi cells, and the stellate/basket cells (Voogd & Glickstein, 1998), and the cerebellar cortex can also be divided into the larger hemispheric and vermal lobules based on the cerebellar fissures (Schmahmann et al., 1999). The classical interpretation of the role of the cerebellum is that it is critical for accurate motor control, prediction, and adaptation (Fine, Ionita, & Lohr, 2002; Glickstein, 2007), consistent with established neuronal connections between the cerebellum and motor cortex via thalamus (Strick, 1985). However, since the late 1980s, evidence suggests that the cerebellum also contributes to a wide range of cognitive processes (Sathyanesan et al., 2019), including attention, executive control, language, working memory, learning, pain, emotion, and addiction (Strick, Dum, & Fiez, 2009). This functional involvement is consistent with anatomical findings of “closed‐looped circuits” between the frontal and parietal cortices of the brain and the cerebellum (Dum & Strick, 2003; Kelly & Strick, 2003; Strick et al., 2009).

Recent works by Bernard et al. (2012) demonstrated the importance of anatomically driven lobular approaches for the resting‐state functional connectivity analysis of the cerebellum. Subsequently, Bernard and Mittal (2014) examined the cerebellar functional topographies in the psychosis population. One critical finding from these studies is the importance of examining cerebellar structure regionally to better understand its role in motor dysfunction in psychosis. However, nonmotor (cognitive) functional relationships between cerebellar lobules and the brain have not been well characterized in psychotic disorders. Consequently, as Bernard et al. suggested, because (a) both motor and nonmotor‐related connectivity changes could exist simultaneously in different cerebellar circuits in schizophrenia, and (b) connectivity analyses from the whole cerebellum could obscure this, an examination of resting‐state functional connectivity based on anatomically segregated cerebellar lobules could provide insight into the cerebellar specific regions and connectivities affected in schizophrenia.

In this study, using anatomically parcellated cerebellar lobules, we examined resting‐state cerebellar–cortical functional connectivity in patients with schizophrenia or schizoaffective disorder and nonpsychiatric control participants. We tested the following three questions: (a) “Does lobular connectivity in the cerebellum differ in schizophrenia?” As found in previous resting‐state studies (Anticevic et al., 2015; Walther et al., 2017), we hypothesized that cerebellar–cortical connectivity in schizophrenia would be increased (i.e., hyperconnectivity) in selective lobules (e.g., Crus I and II as shown in Anticevic et al., 2015) of the cerebellum, depending on the connectivity to the cortical resting‐state functional network of particular lobules as in Walther et al. (2017). (b) “Which functional networks in the cerebrum are predominant in the lobular dysconnectivity of schizophrenia?” In addition to abnormal connectivity in the motor network, typically identified in functional abnormalities in schizophrenia, we also anticipated abnormal connectivity in higher‐level association networks in the cerebrum—for example, ventral attention network as seen in Shinn, Baker, Lewandowski, Ongur, and Cohen (2015) and Guo et al. (2018). Finally, (c) “Is the lobular connectivity associated with motor task behaviors related to cerebellar functioning?” In our previous study, we found that schizophrenia‐spectrum disorders showed abnormal motor‐related effective connectivity during sensorimotor synchronization (Moussa‐Tooks et al., 2019). Therefore, based on the broad overlap between task and resting‐state fMRI abnormalities in schizophrenia (Mwansisya et al., 2017) and the shared cognitive abnormalities in the cortico‐cerebellar circuit in schizophrenia (Sheffield & Barch, 2016), resting‐state cerebellar–cortical functional connectivity was also predicted to be weakly associated with finger‐tapping behavior in individuals with schizophrenia compared to controls. By analogy, in the absence of previous relevant imaging research, the same prediction was made for postural sway, which has been found to be impaired in schizophrenia (Apthorp et al., 2019).

## 2 MATERIALS AND METHODS

### 2.1 Participants

The research protocol was approved by Indiana University's Institutional Review Board for the protection of human subjects. An initial patient sample of 50 was recruited through outpatient and inpatient units at community and state hospitals. Forty‐four healthy control participants were recruited through flyers and advertisements in local publications. Participants provided verbal and written informed consent. Following, demographic, cognitive, handedness (Edinburgh), substance use, and diagnostic details were obtained. Eligible participants were 18 years of age or older, and free of any neurological disorder, head trauma with loss of consciousness greater than 10 min, learning disability, current alcohol/drug abuse or dependence, past alcohol/drug abuse or dependence (6 months for patients; lifetime for controls), current use of illicit substances (confirmed by urine drug screen on the date of MRI), and contraindication to MRI. Clinical interviews and neuropsychological assessments were administered by trained, supervised research personnel in our research center. DSM‐IV‐TR Axis I mood, psychotic, alcohol, and substance abuse/dependence modules of the Structured Clinical Interview for DSM‐IV (SCID‐I; patient version for individuals enrolled as having a psychotic disorder and nonpatient version for individuals enrolled as control participants) were administered. Axis I anxiety disorders were evaluated using the Mini International Neuropsychiatric Interview (MINI). Additionally, participants enrolled with a psychotic disorder were administered the SCID‐II for Axis II personality disorders (i.e., schizoid, schizotypal, paranoid, and antisocial). Control participants did not have any current Axis I disorder and were determined via the Family Interview for Genetic Studies (FIGS) to not have any relatives with a psychotic disorder. After exclusion of participants with excessive head motion during MRI session (see strict criteria in Preprocessing section below), analyses are reported here on 30 individuals with schizophrenia or schizoaffective disorder (for simplicity, referred to throughout as the schizophrenia group) and 37 controls. Final demographic information after the exclusion of ineligible participants is provided in Table 1. The clinical ratings for symptoms of schizophrenia were measured using the Positive and Negative Syndrome Scale (PANSS) for severity of psychopathology (Kay, Fiszbein, & Opler, 1987). Intelligence quotient (IQ) was measured using the Wechsler Abbreviated Scale of Intelligence (WAIS) Full‐Scale Intelligence Quotient (2‐component version; FSIQ‐2; vocabulary and matrix reasoning). Additionally, the Wechsler Adult Intelligence Scale (WAIS) Digit Symbol subscale was administered to evaluate processing speed. Due to the focus of this study on the cerebellum and the association of this structure with a broad range of movement disorders, the International Cooperative Ataxia Rating Scale (ICARS) and the Abnormal Involuntary Movement Scale (AIMS) were administered and Neurological Soft Signs (NSS) were assessed. Antipsychotic medication and chlorpromazine (CPZ) equivalent doses of each patient were evaluated and confirmed through medical records if necessary. All but four patients were taking antipsychotics at the time of study participation (Table S1).

**TABLE 1:** Demographic characteristics for the schizophrenia (SZ) and healthy control participants (HC)

Characteristics	SZ (*n* = 30)	HC (*n* = 37)
Age, mean (SD), years	34.3 (10.4)	38.0 (9.3)
Age, range, years	22–55	20–55
Sex (male/female)^a^	22/8	19/18
Handedness, (R/L/A)^a^ ^,^ ^b^	22/7/1	32/2/3
WASI		
FSIQ^c^	101.13 (15.69)	112.44 (13.79)
Vocabulary^c^	49.80 (12.38)	57.81 (8.66)
Matrix reasoning^c^	53.00 (11.11)	59.97 (13.30)
WAIS diagnosis		
Digit‐symbol^c^	7.70 (2.82)	12.21 (2.89)
Schizophrenia, *n* (%)	21 (70)	
Schizoaffective disorder, *n* (%)	9 (30)	
PANSS		
Positive	13.66 (5.63)	
Negative	13.41 (4.67)	
General	26.72 (9.84)	
Finger‐tapping		
Tone‐paced		
Interval, (ms)	470.15 (41.19)	475.06 (37.50)
CV of interval	0.11 (0.05)	0.09 (0.05)
Self‐paced		
Interval^c^, (ms)	473.77 (56.41)	505.07 (30.76)
CV of interval	0.11 (0.05)	0.08 (0.08)
Postural sway		
Area, log(mm^2^)		
EOOB^c^	1.51 (0.56)	1.15 (0.33)
EOCB^c^	1.64 (0.25)	1.42 (0.23)
ECOB^c^	1.64 (0.46)	1.39 (0.33)
ECCB^c^	1.88 (0.33)	1.67 (0.31)
Path, log(mm)		
EOOB^c^	2.70 (0.23)	2.55 (0.12)
EOCB^c^	2.70 (0.18)	2.57 (0.11)
ECOB^c^	2.78 (0.19)	2.67 (0.14)
ECCB^c^	2.87 (0.19)	2.77 (0.15)

a χ^2^ test.

b Handedness: right/left/ambidextrous.

c Between‐group difference for *p* < .05.

Abbreviations: CV, coefficient of variation; ECCB, eyes‐closed closed‐base; ECOB, eyes‐closed open‐base; EOCB, eyes‐open closed‐base; EOOB, eyes‐open open‐base; FSIQ, Full Scale Intelligent Quotient; WAIS, Wechsler Adult Intelligence Scale; WASI, Wechsler Abbreviated Scale of Intelligence.

### 2.2 Behavioral measures

#### 2.2.1 Finger tapping

Details about the acquisition and processing of finger‐tapping data were described in our previous study (Moussa‐Tooks et al., 2019). Briefly, participants completed three 6‐min, consecutive sessions of a sensorimotor synchronization finger‐tapping task. Each session included six blocks of the following sequence: 6‐s tone‐paced synchronization tapping (with 500‐ms, 2‐Hz pacemaker), 20‐s self‐paced continuation tapping without tone, 6‐s listening, and 15‐s resting periods. Participants tapped with their right index finger on a handheld tapping pad with a 1.5‐cm diameter force sensor. For tone‐paced and self‐paced blocks, mean and variability (defined by the coefficient of variation) of consecutive tapping intervals were measured.

#### 2.2.2 Postural sway

Details about the acquisition and preprocessing of postural sway were described in our previous study (Apthorp et al., 2019). Briefly, participants stood with their eyes either open or closed, and their feet either open or closed, resulting in four conditions, on a force platform for ~2 min. To quantify each sway condition, we measured sway path (path length of the center of pressure during sway trajectory) and area (95% of confidence ellipse computed from two principal axes with sway trajectory; Apthorp, Nagle, & Palmisano, 2014; Duarte & Zatsiorsky, 2002; Tahayor, Riley, Mahmoudian, Koceja, & Hong, 2012). Since the sway path and area were highly skewed, the values were log‐transformed before the analysis.

### 2.3 MRI data acquisition

MRI data were acquired on a Siemens 3T MAGNETOM Trio‐Tim scanner using a 32‐channel head coil. Resting‐state functional scans were collected using an echo‐planar image (EPI) sequence [repetition time (TR) = 700 ms; echo time (TE) = 28 ms; flip angle (FA) = 60°; acquisition matrix = 64 × 64; field of view (FOV) = 220 × 220 mm^2^; in‐plane voxel size = 3.44 × 3.44 × 3.4 mm^3^; 36 transverse slices; no slice gap]. Each participant was instructed to remain still and awake in the scanner, checked by an MRI operator with a subject monitoring camera, and keep their eyes open looking at a fixation‐cross on a screen via an LCD projector, resulting in 1,000 volumes (~12 min). T1‐weighted anatomical scans were collected using an inversion‐recovery spoiled gradient recalled acquisition (IR‐SPGR) sequence [TR = 1.8 s; TE = 2.67 ms; inversion time (TI) = 0.9 s; FA = 9°; acquisition matrix = 256 × 256; in‐plane voxel size = 1 × 1 × 1 mm^3^; 192 sagittal slices].

### 2.4 MRI preprocessing

Functional scans were preprocessed with removal of the first 10 volumes (~7 s), motion parameter estimation, de‐spiking, slice timing correction, motion correction, linear trend removal, within‐run intensity normalization to a whole‐brain mode value of 1,000, linear regression of nuisance variables (see below), and temporal band‐pass filtering (0.009–0.08 Hz). Motion parameter estimation was performed prior to the temporal interpolation of BOLD data (e.g., de‐spiking or slice timing correction), since the temporal interpolation tends to underestimate fMRI motion parameters (Power, Plitt, Kundu, Bandettini, & Martin, 2017). Nuisance parameters for linear regression included six rigid‐body motions, cerebrospinal fluid (CSF), white‐matter (WM), and whole‐brain signals, along with their temporal derivatives, squares, and squares of derivatives (36 regressors; Parkes, Fulcher, Yucel, & Fornito, 2018), in which we applied five erosion cycles to the WM mask and two erosion cycles to the CSF mask to minimize the correlation between WM/CSF and whole‐brain BOLD signal (Parkes et al., 2018; Power, Plitt, Laumann, & Martin, 2017). Functional scans were then co‐registered to each individual's anatomical MRI and normalized to the MNI space. Due to the potential distortion when normalizing the cerebellum (Diedrichsen, Balsters, Flavell, Cussans, & Ramnani, 2009), we separately performed spatial normalization for the cerebral and cerebellar regions (Bernard, Peltier, et al., 2014). The Desikan–Killiany parcellation of Freesurfer (http://www.freesurfer.net) was used to extract the cerebral and cerebellar structures from each anatomical MRI. MNI152 nonlinear T1‐weighted atlas (excluding the cerebellum) and the spatially unbiased atlas template (SUIT; Diedrichsen, 2006; Diedrichsen et al., 2009) were used as templates for the cerebral and cerebellar spatial normalization, respectively. Volumes with high motion in functional scans were censored to minimize potential motion‐artifacts (Power et al., 2014). We used a frame‐wise displacement (FD) threshold of 0.5 mm, assuming 5 cm cortical sphere radius, and a percentage of BOLD signal changes over the whole‐brain (DVARS) of 0.3, above which scans (including 1 backward and 2 forward volumes) were removed (Power, Barnes, Snyder, Schlaggar, & Petersen, 2012). After censoring, the mean FD was <0.5 mm (0.04–0.32) and DVARS <0.15 (0.04–0.11) for all participants. Twenty‐seven participants (20 patients +7 controls) had more than 50% censored scans marked as potentially contaminated by motion artifacts and were excluded from the analyses, resulting in our final sample sized of 30 patients and 37 controls.

### 2.5 Functional connectivity estimation

Spatial smoothing (6‐mm full width at half maximum) in the gray matter mask (eroded by 1 voxel) was applied to the normalized functional scans of cortical regions. BOLD time‐series were extracted in a set of 264 putative brain areas (10‐mm diameter spheres) acquired from a task‐based neuroimaging meta‐analysis (Power et al., 2011). Only the particular set of regions excluding 37 areas—for example, uncertain assignment to a functional system; refer to Power et al. (2011), were selected to represent 10 major functional networks (227 regions; Figure S1). To extract the cerebellar BOLD time‐series, we used 28 lobular parcellation scheme of SUIT cerebellar atlas (Diedrichsen et al., 2009; Figure S1). Since the cortical functional networks are located in the bilateral hemispheres, we accordingly extracted the mean time‐series merging hemispherical regions as well as vermis, which represents 18 lobule‐specific cerebellar BOLD time‐series. Functional scans of each cerebellar lobule are separately extracted, eroded by 1 voxel, and smoothed to minimize BOLD effects from the adjacent cerebellar lobules. Pearson's correlation coefficients were then computed between time series from all pairs of brain regions (227 cortical +18 cerebellar regions) and converted to *z*‐scores using Fisher's *r*‐to‐*z* transformation resulting in 245 × 245 functional connectivity matrix (Figure S2). The global strength of functional connectivity at the *i*‐th cerebellar lobule was defined as follow:

$${\mathrm{FC}}_{i}=\frac{1}{N}\sum_{j=1}^{N}z_{\mathrm{ij}}$$

where *N* represents the number of cortical regions (227) and *z*_*ij*_ the z‐scored functional connectivity from *i*‐th lobule to *j*‐th cortical region. The functional connectivity strength specific to the *k*‐th cortical network was defined as:

$${\mathrm{FC}}_{i}^{k}=\frac{1}{N_{k}}\sum_{j=1}^{N_{k}}z_{\mathrm{ij}}^{k}$$

where *N*_*k*_ represents the number of cortical regions for *k*‐th cortical network.

### 2.6 Statistical analysis

To examine between‐group differences of cerebellar–cortical functional connectivity, we used two‐step approach as used in Anticevic et al. (2015). In detail, each lobular functional connectivity was first correlated with functional connectivities of entire cortical functional network, providing a connectivity map for each participant that was entered into second‐level analyses (i.e., one‐tailed independent samples *t*‐test based on the hypothesis) in which each lobule's value represented its global connectivity with the whole cortices. Subsequently, to examine between‐group differences of the functional connectivity for each cerebellar lobule, all individual FC_*i*_ values were entered into appropriate second‐level tests (i.e., independent samples *t*‐test). Multiple comparison correction was implemented with a false‐discovery rate (FDR) corrected *p* < .05 as the statistical threshold. We included age, sex, and handedness as covariates in this model. Since the patients had more head motion (FD; patients: 0.22 ± 0.09, controls: 0.14 ± 0.08 in mm, *p* < .05), the mean FD for each participant was additionally included as a covariate. Once statistically significant lobules were found, we restricted subsequent analyses to these constrained lobules (Anticevic et al., 2015), showing cerebellar dysconnectivity in the schizophrenia patients, to examine which functional networks are linked to abnormal cerebellar connectivity. Finally, correlational analyses were performed between the functional connectivity measures derived from the restricted subsequent analyses and the finger‐tapping variables (e.g., mean tapping interval and its variability during tone‐ and self‐paced finger‐tapping tasks) and the postural sway variables (e.g., sway path and area for the four sway conditions: eyes‐open open‐base, eyes‐open closed‐base, eyes‐closed open‐base, and eyes‐closed closed‐base).

## 3 RESULTS

### 3.1 Cerebellar–cortical functional connectivity in schizophrenia patients

**FIGURE 1:**
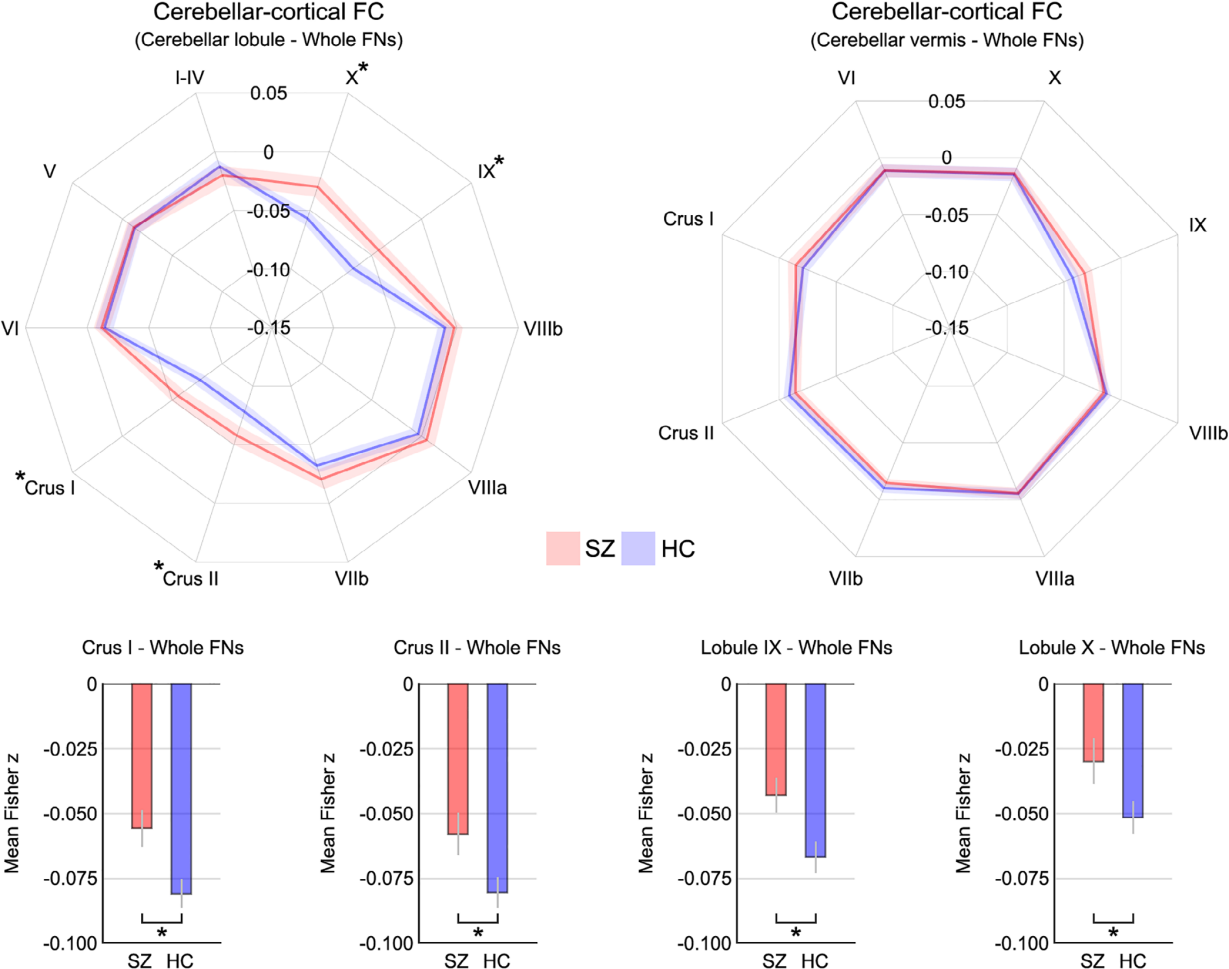
Resting‐state functional connectivity (FC) from the cerebellum (10 hemispheric lobules +8 vermal regions) to the whole cortical functional networks (FN) described in Power et al. (2011). Between‐group differences (* marks) were found for four tentative regions of interest in the cerebellar hemispheres—Crus I, lobule IX, and lobule X with false‐discovery rate (FDR) corrected *p* < .05; Crus II with uncorrected *p* < .05. The scale of radar plots ranged from −0.15 to 0.05 using increments of 0.05. Red and blue colors indicate patients with schizophrenia (SZ) and healthy control participants (HC), respectively. Shaded areas and error bars represent ±1 SEM

**FIGURE 2:**
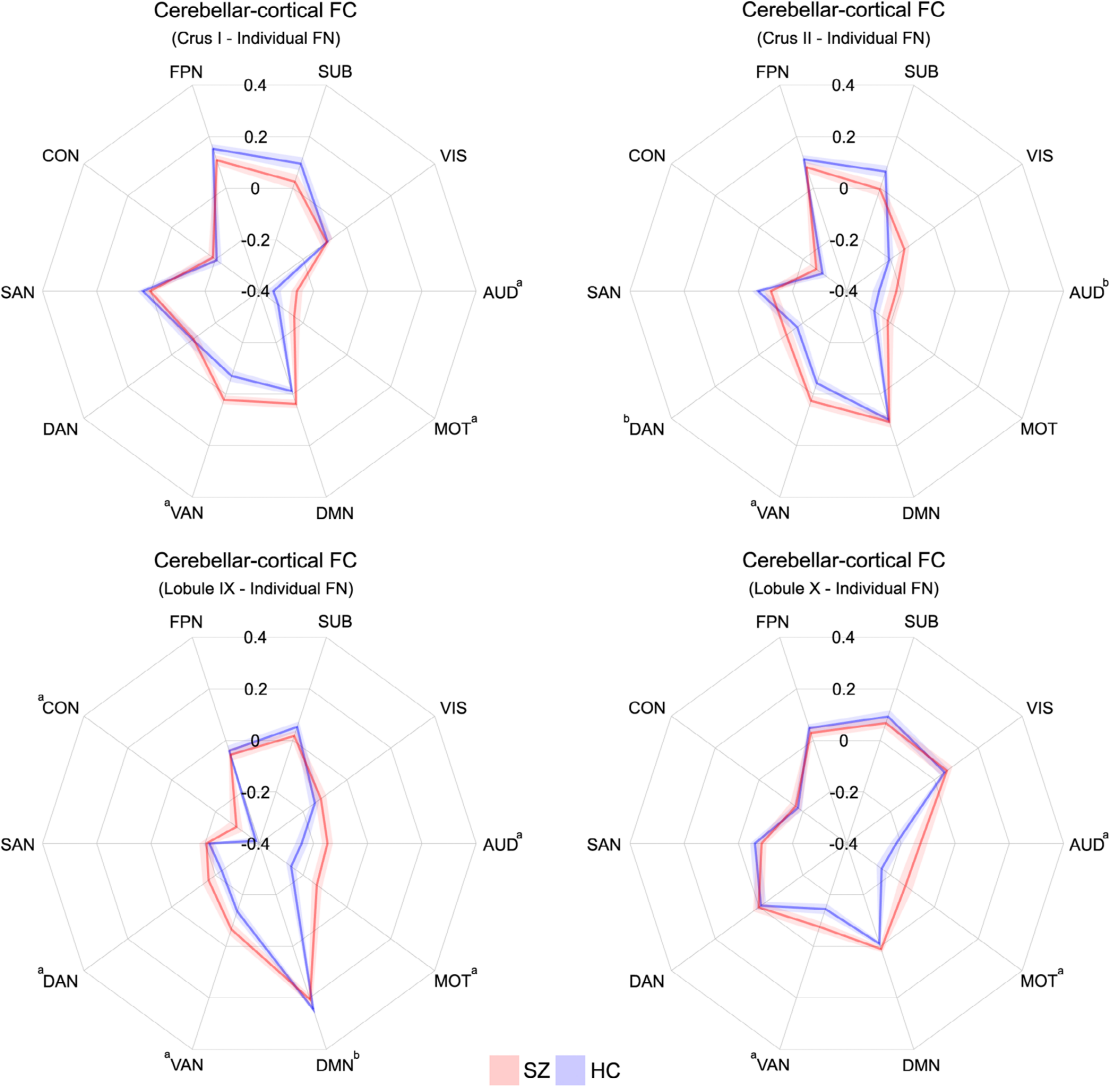
Resting‐state functional connectivity (FC) from 4 tentative cerebellar lobules found in Figure 1 to the individual cortical functional networks (FN) described in Power et al. (2011). ^a^False‐discovery rate (FDR) corrected *p* < .05. ^b^Uncorrected *p* < .05. The scale of radar plots ranged from −0.4 to 0.4 using increments of 0.2. Red and blue colors indicate patients with schizophrenia (SZ) and healthy control participants (HC), respectively. Shaded areas represent ±1 SEM. Abbreviations: AUD, auditory network; CON, cingulo‐opercular network; DAN, dorsal attention network; DMN, default‐mode network; FPN, frontoparietal network; MOT, motor and somatosensory network; SAN, salience network; SUB; subcortical network; VAN, ventral attention network; VIS, visual network

We first tested whether the cerebellar–cortical functional connectivity from each cerebellar lobule differed between the schizophrenia and control groups. In a two‐step process as used in a previous study (Anticevic et al., 2015), we initially examined functional connectivity between the 18 cerebellar lobules and the whole‐cerebrum with 10 cortical functional networks (Figure S1). Between‐group differences were found in the cerebellar–cortical functional connectivity from Crus I, lobule IX, and lobule X (FDR‐corrected *p* < .05 with Cohen's *d*: .60–.65) and a trend‐level difference in Crus II (*p* = .01 with Cohen's *d* = .50, but not surviving FDR‐correction) to the whole cortical functional networks (Figure 1 and Table S2). In the second step, we subsequently examined the connectivity of these four cerebellar lobules with the 10 cortical functional networks; Crus II was included due to the potential abnormalities found in previous schizophrenia resting‐state studies (Ding et al., 2019; Gao et al., 2019; Guo et al., 2015; Shinn et al., 2015). Our analyses revealed robust between‐group differences (FDR‐corrected *p* < 0.05) for the specific cortical functional networks (Figure 2 and Table S3). Marked connectivity increases (i.e., hyperconnectivity) for the schizophrenia patients compared to controls were observed: (a) between Crus I and the ventral attention, motor, and auditory networks; (b) between Crus II and the ventral attention network; (c) between lobule IX and the ventral and dorsal attention, motor, auditory, and cingulo‐opercular networks; and (d) between lobule X and the ventral attention, motor, and auditory networks, consistent with previous observations of cortical network abnormalities in schizophrenia (Bernard & Mittal, 2014; Dong, Wang, Chang, Luo, & Yao, 2018; Jimenez et al., 2016; Wynn et al., 2015). Hyperconnectivity, which might also be seen as less anti‐correlation, was present even using a methodological adjustment, global‐signal regression (Figure S3–S5). No vermal regions showed functional connectivity changes in the patients (Figure S6).

### 3.2 Behavioral performance of finger‐tapping and postural sway

During the self‐paced finger‐tapping task, individuals with schizophrenia had shorter tapping intervals compared to control groups (patients: controls = 473.77 ± 56.41:505.07 ± 30.76 in ms; *t* = 2.54, *p* = .01, Cohen's *d* = .68; Figure S7), while the patients during the same task showed a trend‐level increase of tapping variability measured by the coefficient of variation (CV) of tapping intervals (patients: controls = 0.11 ± 0.05:0.08 ± 0.08 in ms; *t* = 1.73, *p* = .09, Cohen's *d* = .48). Also, the sway path and area for individuals with schizophrenia were significantly greater than for control groups (*p* < .05 for all conditions). Findings of finger‐tapping and postural sway measures were consistent with our previous studies (Apthorp et al., 2019; Moussa‐Tooks et al., 2019).

### 3.3 Association between finger‐tapping and cerebellar–cortical connectivity

**FIGURE 3:**
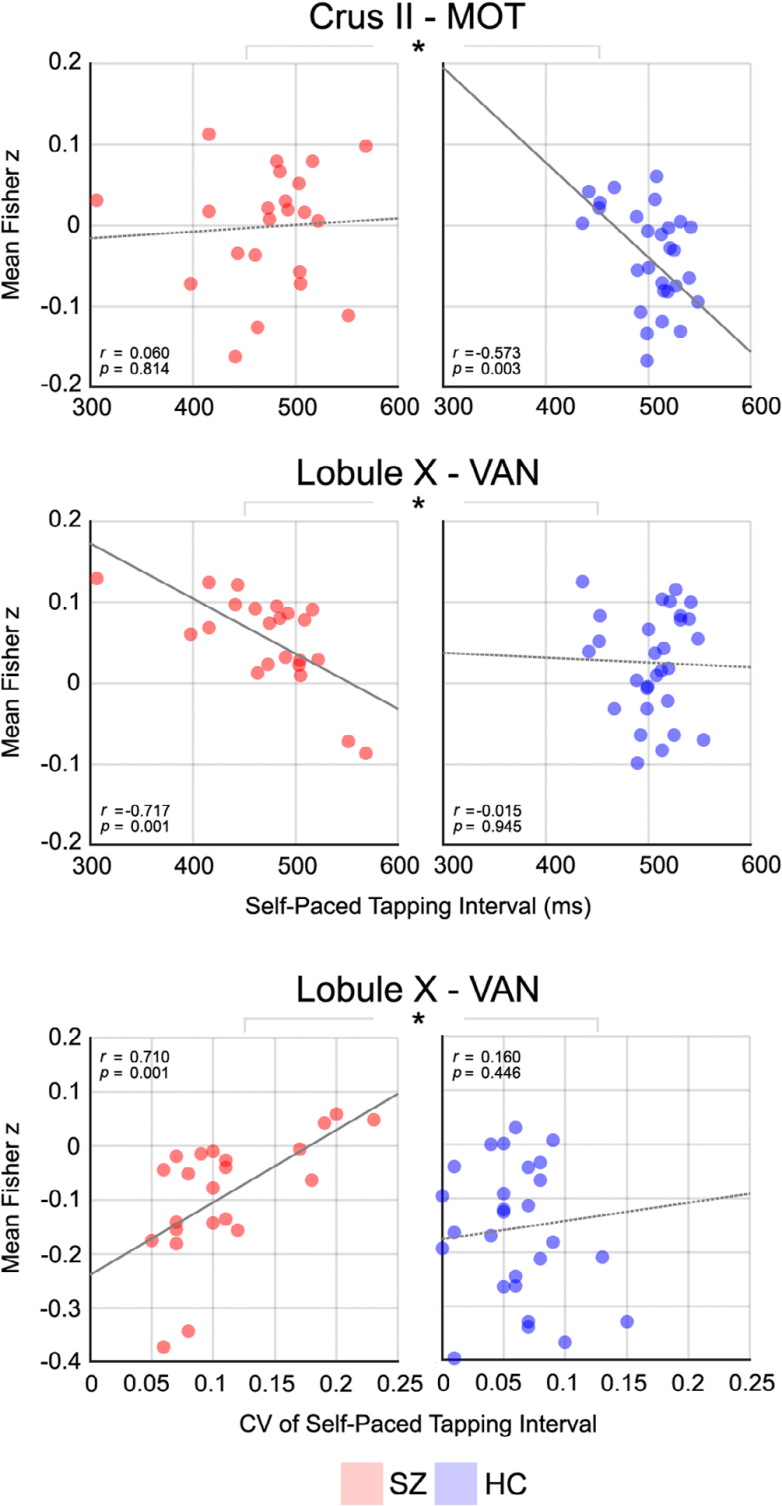
Significant associations between cerebellar–cortical functional connectivity (FC) and finger‐tapping behavioral measures. Correlations were evaluated only in four altered lobules found in Figures 1 and 2, significant associations in each group (*p* < .05) were indicated with gray solid lines, and between‐group effects (*marks) between correlation coefficients of two groups at *p* < .05 (Fisher's *z*‐test)

First, we correlated finger‐tapping measures (i.e., tapping intervals and variability) with functional connectivity values separately for the four constrained lobules found in the functional connectivity analysis above (i.e., Crus I, Crus II, lobule IX, and lobule X), and then subsequent post hoc exploratory analyses were also performed in the other cerebellar lobules to investigate potential resting‐task associations. In patients, significant correlations were observed between higher functional connectivity in lobule X to the whole functional network, but most prominently in ventral attention network, and shorter (*r* = −.717, *p* = .001, FDR‐corrected) and more variable (*r* = .710, *p* = .001, FDR‐corrected) tapping intervals (Figure 3). No such associations were found in the control group. Relatedly, the associations of functional connectivity between lobule X and the ventral attention network showed significant between‐group differences (Fisher's *z*‐test: *p* < .05). In the control group, individuals with less cerebellar–cortical connectivity (i.e., between the Crus II and the whole functional network, but most prominently the motor network) had longer self‐paced tapping intervals (*r* = −.573, *p* = .003, FDR‐corrected), while the patients had dissociable connectivity patterns (*r* = .060, *p* = .814 for the Crus II and motor network) between the tapping behavior and functional connectivity. Associations from the cerebellar lobules to the whole cortical functional networks were shown in Figure S8. Of note, we did not identify a significant association between identified cerebellar–cortical dysconnectivity and the tone‐paced tapping measures.

### 3.4 Association between postural sway and cerebellar–cortical connectivity

**FIGURE 4:**
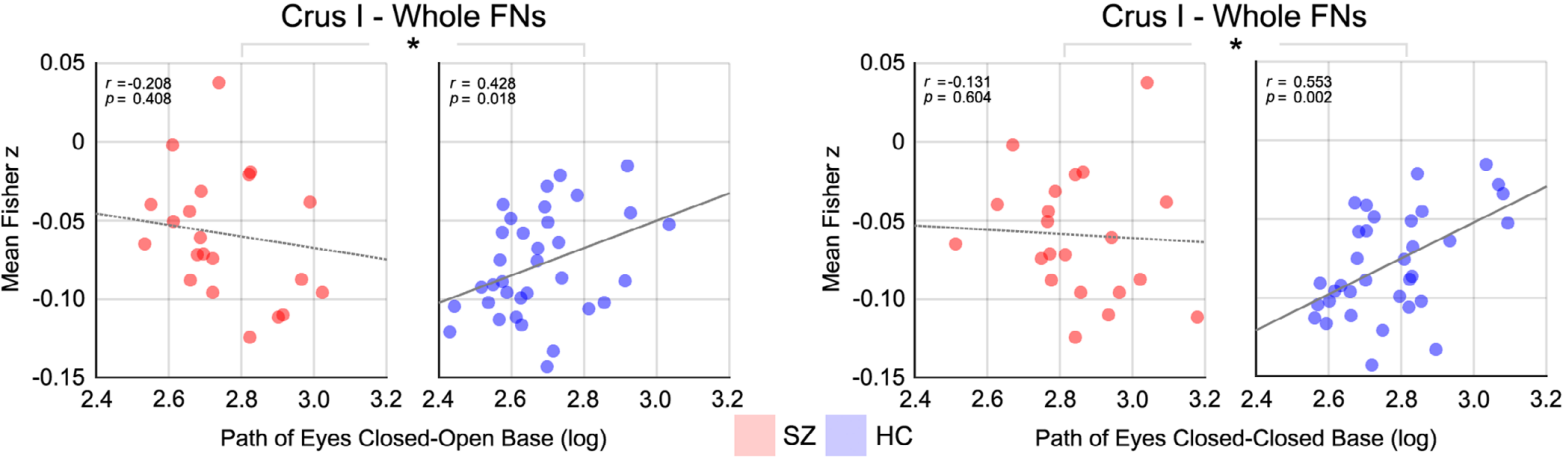
Significant associations between cerebellar–cortical functional connectivity (FC) and postural sway measures. Correlations were evaluated only in four altered lobules found in Figures 1 and 2, and significant associations (*p* < .05) found in healthy control participants (HC) were indicated with gray solid lines. *Between‐group effects between correlation coefficients of two groups at *p* < .05 (Fisher's *z*‐test)

There were also significant positive associations between the sway path of eyes‐closed conditions and functional connectivity of the Crus I across the control participants (eyes‐closed open‐base: *r* = .428, *p* = .018; eyes‐closed closed‐base *r* = .553, *p* = .002; FDR‐corrected; Figure 4) with no associations in patients, which showed significant between‐group differences (Fisher's *z*‐test: *p* < .05).

## 4 DISCUSSION

In this study, lobule‐specific resting‐state connectivity analysis in the cerebellum revealed that (a) selective lobules in the cerebella of individuals with a schizophrenia‐spectrum disorder exhibit increased cerebellar–cortical functional connectivity, particularly in Crus I, Crus II, lobule IX, and lobule X; (b) hyperconnectivity was most robust for connections to the ventral attention network, thought to support attentional filtering (Vossel, Geng, & Fink, 2014), as well as the motor and auditory networks; and (c) cerebellar–cortical connectivity in the resting‐state was differentially associated with sensorimotor task‐based behavioral measures (i.e., finger‐tapping and postural sway) in schizophrenia compared to controls. These results provide evidence of aberrant linkage between specific cerebellar lobules and resting‐state networks (sensory, motor, and attentional), lending support to more recent conceptualizations of the cerebellum's importance in coordinating a wide range of cognitive and regulatory processes beyond motor function. Moreover, impairment within these networks might contribute to the cerebellar motor and cognitive deficits observed in individuals with schizophrenia‐spectrum disorders, as well as those at‐risk for psychosis (Bernard & Mittal, 2014).

### 4.1 Cerebellar–cortical functional connectivity in schizophrenia patients

Previous studies examining resting‐state functional connectivity in the cerebellum demonstrated alterations of the cerebellar association with cortical networks of schizophrenia, particularly in the somatomotor system. For example, motivated by the cortico‐cerebellar‐thalamo‐cortical model to elucidate functional involvement of the cerebellum, Cao et al. found persons with schizophrenia exhibit broad cerebellar functional hyperconnectivity at rest and while engaged in cognitive tasks, which covers the nonmotor cortical subnetworks (e.g., frontoparietal, attention, and default‐mode networks) as well as the sensorimotor cortices (e.g., precentral, postcentral, and supplementary motor areas), suggesting a state‐independent neural trait in schizophrenia (Cao et al., 2018). While Cao et al. showed abnormal functional connectivity between the cerebellum and cortical areas (i.e., both sensorimotor and frontotemporal regions) in schizophrenia, their cerebellar seed regions of interest were located mostly at the anterior parts of the cerebellum based on Power's brain atlas (i.e., lobule V and VI; Power et al., 2011), suggesting potential associations between sensorimotor lobules of the cerebellum and the cortical association networks. Here, we demonstrated consistent hyperconnectivity from the cerebellum to cortex in schizophrenia, but specifically for lobules in Crus I, Crus II, lobule IX, and lobule X, which are known for their functional role in the cognitive processes (Figures 1 and 2).

In this study, the most prominent connectivity alterations in schizophrenia were observed between the cerebellum and the ventral attention network (Figure 2). The ventral attention network is involved in reorienting attention in response to salient sensory stimuli (Fox, Corbetta, Snyder, Vincent, & Raichle, 2006), resulting in the increased functional activities for the detection of changes in the sensory environment (Downar, Crawley, Mikulis, & Davis, 2000). There is also converging evidence of connectivity abnormalities in this functional network in schizophrenia, suggesting that the ventral attention network may play a core role in the disconnected large‐scale brain networks with self‐representation and environmental salience processing of schizophrenia (Dong et al., 2018). Our finding of cerebellar functional dysconnectivity to the ventral attention network, therefore, suggests that patients may be using compensatory adaptation to maintain intrinsic baseline resting‐state—for example, consciousness, monitoring body position, processing sensory and automatic signals, tracking the passage of time, and so on (Binder et al., 1999).

Previous findings, however, have been somewhat controversial in terms of direction of the connectivity difference. While increased functional connectivity in schizophrenia was found at rest with frontoparietal regions including the ventral attention network associated with cognitive deficits (Anhoj et al., 2018), Dong et al. demonstrated, in individual with schizophrenia, hypoconnectivities between the ventral attention network and other cortical regions with the default‐mode, frontoparietal, and subcortical networks (Dong et al., 2018). In the cerebellar resting‐state analysis, similar hypoconnectivity in schizophrenia was often found between the ventral attention network and the corresponding cerebellar clusters (e.g., Crus II and vermis VI; Shinn et al., 2015) and a seed voxel in the lobule VI (Guo et al., 2018). On the other hand, we found increased functional connectivity from the cerebellum (particularly, Crus I, Crus II, lobule IX, and lobule X) to the ventral attention network in schizophrenia. In addition to the complicated nature of functional alterations in the cerebellum of schizophrenia, directional inconsistency of the connectivity difference, in part, may be due to the different selection strategies of the cerebellar seed regions for the computation of functional connectivity. Specifically, Shinn et al. (2015) and Guo et al. (2018) tried to find the most prominent cortico‐cerebellar connections at the whole or the limited set of voxel‐level defined by the functional parcellation (Buckner et al., 2011), and the findings overlapped with different cortical networks even in the same cerebellar lobule. For instance, Crus II in schizophrenia had both hypoconnectivities to the ventral attention, salience, control, and default‐mode networks, and hyperconnectivities to the somatomotor and another default‐mode networks (Shinn et al., 2015), which suggests a diverse functional role and heterogenous connectivity profiles in anatomically defined cerebellar lobules (Bernard et al., 2012; Bernard & Mittal, 2014). Accordingly, biological meaning of our results in the ventral attention network should be cautiously interpreted. Importantly, our results suggest that functional efficiency, not functional activity itself, was impaired between the cerebellum and ventral attention network of schizophrenia, which may be reflected in these group differences in functional connectivity (Sun, Collinson, Suckling, & Sim, 2019).

While it is noteworthy that alterations in the direction of resting‐state sensorimotor functional connectivity have been observed in the cerebella of individuals with schizophrenia, increased (Cao et al., 2018) or decreased (Anticevic et al., 2015; Berman et al., 2016) hyperconnectivity of the cerebellum to the sensorimotor system has been more commonly reported (Bernard, Goen, & Maldonado, 2017; Cao et al., 2018; Shinn et al., 2015; Walther et al., 2017). For instance, Walther et al. showed hyperconnectivities within the resting‐state motor network in schizophrenia, which consisted of higher cortico‐cerebellar and thalamo‐cortical connections, but not the cerebellar‐thalamo connection (Walther et al., 2017). In addition, Ji et al. (2019) found cerebellar hyperconnectivity, in particular, with somatomotor and superior temporal cortices in schizophrenia, suggesting the disruption of large‐scale cortico‐striatal‐thalamic‐cerebellar functional pathways (Anticevic et al., 2015). Our results are in line with these previous findings that cerebellar hyperconnectivity is prominent in the cortical motor network in schizophrenia. As with Ji et al. (2019), we also found hyperconnectivity in the cortical auditory network. While Buckner's initial study suggested that the primary visual and auditory cortices may not be functionally connected to cerebellum at rest (Buckner et al., 2011), they also recognized the possibility that the cerebellar regions might be connected to their second dominant principal cerebral targets. Our hyperconnectivity finding with the auditory network may suggest a potential auditory contribution of the cerebellum in schizophrenia, which is supplementary to the primary sensorimotor function.

### 4.2 Association between finger‐tapping and cerebellar–cortical connectivity

In our previous study, we demonstrated that self‐paced tapping, in which the cerebellum is highly involved in the temporal coordination of motor activities, is impaired in schizophrenia, and showed a dyscoordination of the cortico‐cerebellar‐thalamo‐cortical circuit during this task (Moussa‐Tooks et al., 2019). The current study demonstrated that resting‐state connectivity from Crus I and II (cognitive‐related) was disproportionally associated with cerebellar‐mediated sensorimotor performance in patients with schizophrenia during the self‐paced portion of the task. In healthy participants, tapping intervals further from the target interval (i.e., reflecting poorer task performance) were correlated with decreased cerebellar connectivity in Crus II (Figure 3) suggesting a comparable cerebellar coordination of the nonmotor processes (Andreasen & Pierson, 2008). Patients, however, demonstrated an absence of these associations; instead, the cerebellar connectivity from lobule X to the ventral attention network were negatively correlated with the tapping interval and positively with tapping variability. Lobule X, the substrate of the vestibulocerebellum, is known to be involved in head and body orientation information processing to help maintain balance (Johns, 2014) and executive functions such as attentional set‐shifting and cognitive inhibition (T. Kim et al., 2018), and it is also known to be functionally connected to the default‐mode network (Bernard & Mittal, 2014). The self‐paced portion of sensorimotor synchronization has been shown to more strongly engage temporal processing functions, compared to tone‐paced synchronization, for which the current study found no correlations. Accordingly, these observed correlations together suggest the broader coordinative role that the cerebellum contributes across critical domains (i.e., motor, sensory, and attentional) while at rest or engaged in a task. Specific to schizophrenia, observed associations between poorer tapping performance and cerebellum to ventral attention network hyperconnectivity may indicate: (a) a neural compensatory mechanism in an attempt to remediate poor cerebellar error‐correction processes and/or (b) a deficit in behavioral performance as a function of excess neural signal.

### 4.3 Association between postural sway and cerebellar–cortical connectivity

Postural sway is known to be sensitive to sensorimotor functioning, which includes the cerebellum, and impaired postural control as determined by increased postural sway in psychosis (e.g., ultra high‐risk of psychosis) has been suggested as a potential biomarker (Bernard, Dean, et al., 2014). Postural sway is highly dependent on sensory integration of vestibular, visual, and proprioceptive inputs as well as the basal ganglia and motor cortices (Apthorp et al., 2019; Jacobs & Horak, 2007). We found that cerebellar lobular connectivity to cortical resting‐state networks was differentially associated with postural sway depending on group (Figure 4). Specifically, in healthy participants, less sway (e.g., “better” task performance as indicated by smaller area and shorter path length of sway) was associated with lower connectivity from the nonmotor cerebellar lobule (i.e., Crus I), as would be expected during a fundamentally motor task. Patients, however, had more sway (“worse” task performance) and did not show such associations in the nonmotor cerebellar lobule. This finding of impaired cerebellar rest‐task association in schizophrenia is largely consistent with a previous postural sway study demonstrating abnormal cerebellar connectivities to both motor and nonmotor cortices in individuals at ultra‐high‐risk for the development of psychosis (Bernard, Dean, et al., 2014). Accordingly, our finding is indicative of altered interactions between the cerebellum and cortical networks, in which sensorimotor performance might have an altered association with cerebellar resting‐state connectivity at specific lobules in schizophrenia.

### 4.4 Pathophysiological implications

Alterations in functional connectivity might reflect anatomical or neurophysiological alterations within cellular networks. Anatomically, there is evidence of reduction of dendritic spine density, particularly affecting pyramidal cells, which would affect long range signaling (Glausier & Lewis, 2013). From a neurophysiological perspective, NMDA receptor dysfunction has been implicated in the pathophysiology of schizophrenia (Javitt, Zukin, Heresco‐Levy, & Umbricht, 2012). In humans, acute administration of the NMDA receptor antagonist, ketamine, elicits symptoms and cognitive deficits characteristic of schizophrenia. Similarly, in rodent models, ketamine and other NMDA receptor antagonists can produce behavioral and electrophysiological deficits similar to those observed in schizophrenia, such as working memory deficits and reduction of the mismatch negativity of the event‐related potential. It is intriguing in light of these data that ketamine administration in humans frequently produces functional hyperconnectivity on MR imaging (Maltbie, Kaundinya, & Howell, 2017), similar to the findings in the current study.

### 4.5 Limitations

This study has several limitations. First, medication assessed by chlorpromazine‐equivalent doses in this study was not correlated with any sensorimotor behavioral or functional connectivity measures. However, the antipsychotic medications might affect the resting‐state connectivity of some functional networks in the cortical areas (e.g., dorsal attention, executive control, and salience networks; Kraguljac, White, Hadley, Visscher, et al., 2016) as well as subcortical regions—for example, the striatum (Sarpal et al., 2015), hippocampus (Kraguljac, White, Hadley, Hadley, et al., 2016), and ventral tegmental area (Hadley et al., 2014). Antipsychotic medication has been known to be associated with motor dysfunction in schizophrenia (Peralta, Campos, De Jalon, & Cuesta, 2010; Whitty, Owoeye, & Waddington, 2009). Accordingly, we cannot rule out the possibility that the medication effect influences altered cerebellar–cortical connectivities in schizophrenia in ways that are not detected by the current samples. Several points mitigate this concern: (a) Woodward, Karbasforoushan, and Heckers (2012) showed that the prefrontal–thalamic and motor/somatosensory–thalamic connectivity, which are core constituents of the cerebro‐cerebellar loops, were not related to antipsychotic medication dosage in schizophrenia. (b) Drug‐naïve schizophrenia patients showed functional hyperconnectivity between the thalamus and the sensorimotor cortex—that is, a part of the cortico‐cerebellar‐thalamo‐cortical loop (Martino et al., 2018), comparable to the hyperconnectivty between the cerebellum and the motor functional network in the patients of this study. (c) Antipsychotic medication may both improve pre‐existing abnormalities and cause “de novo” neurologic syndromes (Peralta & Cuesta, 2010). (d) It is important to note that in our sample, ratings of involuntary motor activity (i.e., ICARS, AIMS, and NSS) did not correlate with connectivity measures. Second, it is possible that the different diagnostic categories or clinical features may be differentially associated with the functional connectivity changes or the connectivity‐behavior associations in the cerebellum. Third, we limited our cerebellar lobule‐specific connectivity analyses only to those lobules that showed group differences in connectivity with whole‐brain cortical connectivity to reduce multiple comparisons. Given the reduced power of the present study, our findings warrant further examination with larger samples. The relatively small sample size limited our ability to assess the impact of additional variables (e.g., clinical status, chlorpromazine‐equivalent doses, etc.). In addition, the exploratory nature of the analyses of the behavioral correlates warrants caution until larger studies are conducted wherein more stringent corrections for multiple comparisons can be appropriately applied. Forth, the resting‐state acquisition period in this study was relatively short (~12 min), especially compared to recent recommendations for much longer sessions (Marek et al., 2018), which may be impractical for clinical studies due to head motion.

## 5 CONCLUSION

Significant functional hyperconnectivity was found between specific cerebellar lobules and cortical resting‐state networks in schizophrenia. Moreover, cerebellar functional connectivity of both motor and nonmotor lobules to cortical networks was associated with impaired sensorimotor synchronization and postural control in schizophrenia. These connectivity alterations in the cerebellum are consistent with dysconnectivity theories of schizophrenia (Andreasen & Pierson, 2008), and expand understanding of the role of the cerebellum beyond motor function to include broader cerebral coordinative processes. Findings of cerebellar hyperconnectivity and abnormal connectivity associations with sensorimotor behavioral tasks suggest that lobule‐specific resting‐state functional connectivity may be a candidate biological marker of schizophrenia and may predict a putative relationship with behavioral mechanisms of motor and cognitive processes.

## Supporting information


**Appendix S1** Supplementary Information

## Data Availability

The data that support the findings of this study are available from the corresponding author upon reasonable request.
