# Supplementary material for: Cerebellar–cortical dysconnectivity in resting‐state associated with sensorimotor tasks in schizophrenia
Source: Hum Brain Mapp. 2020 Apr 6;41(11):3119–32. doi: 10.1002/hbm.25002 (PMC7336143; doi:10.1002/hbm.25002)
Supplement: Supplementary file 1 — Appendix S1 Supplementary Information [file HBM-41-3119-s001.docx]

**Effects of Global Signal Regression**

The impact of global signal regression has been debated in resting-state functional connectivity analysis (Liu, Nalci, & Falahpour, 2017; Murphy, Birn, Handwerker, Jones, & Bandettini, 2009; Murphy & Fox, 2017), but the global BOLD signal is known to be largely related to participant motion, particularly, due to head movement during respiration (Power et al., 2018). Since the inclusion of global signal regression as a preprocessing step prior to analyses is known to shift the distribution of correlations around zero (i.e., centering), physiological interpretation of negative correlations has often been difficult. However, a recent consensus in the resting-state fMRI analysis is to use global signal regression to minimize the potential effect of participant head motion (Murphy & Fox, 2017); therefore, we initially included it as a preprocessing step. On the other hand, there is emerging evidence that the topography of global signal is spatially altered in schizophrenia patients (Yang et al., 2017; Yang et al., 2014). Therefore, we re-tested our results to check if the findings were maintained without global signal regression. As expected, functional connectivity values with and without global signal regression were positively correlated (*r* > 0.7, *p* < 0.001; Supplementary Figure 3), demonstrating higher connectivity values with global signal regression also tend to have higher connectivity values even without global signal regression (i.e., shifting). While the functional connectivities without global signal regression were distributed on the positive axis, the patients had consistent hyperconnectivity, which was consistent with our results when removing the global signal. Analyses without global signal regression, however, didn’t show any significant between-group differences, possibly due to less sensitivity to reduce motion-related artifact in resting scans (Supplementary Figure 4-5). Therefore, we reported our results using global signal regression.

**Table 1.** Medication details for the schizophrenia (SZ) samples.

| Medication | SZ  (*n* = 30) |
| --- | --- |
| Antipsychotic-free, No. (%) | 4 (13) |
| Typical antipsychotic, No. (%) | 5 (17) |
| Haloperidol | 2 (7) |
| Fluphenazine | 2 (7) |
| Thiothixene | 1 (3) |
| Atypical antipsychotic, No. (%) | 23 (77) |
| Clozapine | 5 (17) |
| Paliperidone | 5 (17) |
| Quetiapine | 5 (17) |
| Olanzapine | 4 (13) |
| Risperidone | 2 (7) |
| Aripiprazole | 2 (7) |
| Asenapine | 2 (7) |
| Lurasidone | 1 (3) |
| Antidepressant, No. (%) | 10 (33) |
| Benzodiazepine, No. (%) | 4 (13) |
| Lithium, No. (%) | 3 (10) |
| Antiepileptic, No. (%) | 2 (7) |

**Table 2.** Cerebellar-cortical functional connectivity from each cerebellar lobule to the whole cortical functional networks.

| Regions | | SZ (*n* = 30) | HC (*n* = 37) | *t*-value | *p*-value |
| --- | --- | --- | --- | --- | --- |
| Hemisphere | I-IV | -0.020 (0.045) | -0.013 (0.034) | -0.31 | 0.62* |
|  | V | -0.011 (0.035) | -0.012 (0.037) | 0.61 | 0.27* |
|  | VI | -0.012 (0.035) | -0.014 (0.045) | 0.60 | 0.27* |
|  | Crus I | -0.056 (0.037) | -0.078 (0.034) | 2.75 | < 0.001* |
|  | Crus II | -0.058 (0.043) | -0.078 (0.037) | 2.34 | 0.01^†^ |
|  | VIIb | -0.021 (0.046) | -0.032 (0.033) | 0.88 | 0.19* |
|  | VIIIa | 0.005 (0.044) | -0.003 (0.040) | 0.86 | 0.80* |
|  | VIIIb | -0.002 (0.039) | -0.010 (0.036) | 0.69 | 0.24* |
|  | IX | -0.043 (0.035) | -0.068 (0.040) | 2.55 | < 0.001* |
|  | X | -0.030 (0.046) | -0.057 (0.034) | 2.84 | < 0.001* |
| Vermis | VI | -0.011 (0.031) | -0.011 (0.036) | 0.44 | 0.33* |
|  | Crus I | -0.015 (0.038) | -0.021 (0.032) | 0.72 | 0.24* |
|  | Crus II | -0.014 (0.027) | -0.009 (0.031) | -0.29 | 0.61* |
|  | VIIb | -0.015 (0.018) | -0.010 (0.024) | -0.97 | 0.83* |
|  | VIIIa | -0.006 (0.028) | -0.005 (0.031) | -0.08 | 0.53* |
|  | VIIIb | -0.015 (0.022) | -0.013 (0.025) | -0.91 | 0.82* |
|  | IX | -0.032 (0.036) | -0.042 (0.034) | 1.61 | 0.06* |
|  | X | -0.014 (0.028) | -0.014 (0.037) | 0.38 | 0.35* |

*Abbreviations*: SZ, schizophrenia patients; HC healthy controls. *Between-group difference, FDR-corrected *p* < 0.05, and †uncorrected *p* < 0.05.

**Table 3.** Cerebellar-cortical functional connectivity from the constrained cerebellar lobules showing cerebellar-cortical dysconnectivity in the patient group to the cortical functional networks.

| Cerebellar Lobule | Cortical  Functional Network | SZ  (*n* = 30) | HC  (*n* = 37) | *t*-value | *p*-value |
| --- | --- | --- | --- | --- | --- |
| Crus I | FPN | 0.109 (0.110) | 0.153 (0.106) | -1.97 | 0.05* |
|  | CON | -0.189 (0.113) | -0.206 (0.141) | 0.78 | 0.44* |
|  | SAN | 0.003 (0.106) | 0.029 (0.122) | -1.25 | 0.22* |
|  | DAN | -0.099 (0.111) | -0.095 (0.149) | -0.04 | 0.97* |
|  | VAN | 0.022 (0.093) | -0.071 (0.129) | 4.09 | < 0.001* |
|  | DMN | 0.038 (0.095) | -0.012 (0.092) | 1.87 | 0.07* |
|  | MOT | -0.241 (0.142) | -0.314 (0.144) | 2.66 | < 0.01* |
|  | AUD | -0.261 (0.114) | -0.348 (0.163) | 3.25 | < 0.01* |
|  | VIS | -0.092 (0.142) | -0.088 (0.124) | -0.35 | 0.73* |
|  | SUB | 0.025 (0.149) | 0.095 (0.151) | -1.69 | 0.10* |
| Crus II | FPN | 0.081 (0.125) | 0.112 (0.098) | -0.56 | 0.58* |
|  | CON | -0.261 (0.148) | -0.290 (0.127) | 1.43 | 0.16* |
|  | SAN | -0.120 (0.125) | -0.073 (0.137) | -0.98 | 0.33* |
|  | DAN | -0.125 (0.134) | -0.174 (0.129) | 2.17 | 0.03* |
|  | VAN | 0.026 (0.115) | -0.043 (0.128) | 2.89 | < 0.01* |
|  | DMN | 0.107 (0.127) | 0.099 (0.106) | -0.34 | 0.74* |
|  | MOT | -0.213 (0.146) | -0.274 (0.135) | 1.86 | 0.07* |
|  | AUD | -0.217 (0.147) | -0.281 (0.143) | 2.18 | 0.03* |
|  | VIS | -0.137 (0.127) | -0.208 (0.125) | 1.99 | 0.05* |
|  | SUB | -0.004 (0.140) | 0.064 (0.140) | -1.47 | 0.15* |
| IX | FPN | -0.056 (0.122) | -0.040 (0.120) | 0.72 | 0.47* |
|  | CON | -0.296 (0.162) | -0.385 (0.111) | 3.16 | < 0.01* |
|  | SAN | -0.205 (0.145) | -0.215 (0.131) | 0.67 | 0.50* |
|  | DAN | -0.169 (0.142) | -0.229 (0.110) | 3.12 | < 0.01* |
|  | VAN | -0.066 (0.120) | -0.136 (0.123) | 2.54 | 0.01* |
|  | DMN | 0.206 (0.133) | 0.245 (0.095) | -2.23 | 0.03* |
|  | MOT | -0.138 (0.124) | -0.255 (0.140) | 3.14 | < 0.01* |
|  | AUD | -0.148 (0.109) | -0.244 (0.144) | 2.84 | < 0.01* |
|  | VIS | -0.119 (0.130) | -0.147 (0.140) | 0.45 | 0.66* |
|  | SUB | 0.017 (0.113) | 0.052 (0.126) | -0.64 | 0.52* |
| X | FPN | 0.029 (0.107) | 0.048 (0.088) | -0.55 | 0.59* |
|  | CON | -0.167 (0.132) | -0.177 (0.109) | 0.71 | 0.48* |
|  | SAN | -0.086 (0.098) | -0.061 (0.104) | -0.61 | 0.55* |
|  | DAN | 0.002 (0.137) | -0.009 (0.143) | 0.39 | 0.70* |
|  | VAN | -0.076 (0.108) | -0.145 (0.119) | 2.59 | 0.01* |
|  | DMN | 0.010 (0.072) | -0.010 (0.069) | 1.16 | 0.25* |
|  | MOT | -0.132 (0.165) | -0.241 (0.141) | 2.71 | 0.01* |
|  | AUD | -0.128 (0.141) | -0.217 (0.138) | 2.68 | < 0.01* |
|  | VIS | 0.057 (0.143) | 0.046 (0.116) | 0.02 | 0.98* |
|  | SUB | 0.067 (0.110) | 0.092 (0.143) | -0.53 | 0.60* |

*Abbreviations*: FPN, fronto-parietal network; CON, cingulo-opercular network; SAN, salience network; DAN, dorsal attention network; VAN, ventral attention network; DMN, default-mode network; MOT, motor and somatosensory network; AUD, auditory network; VIS, visual network; SUB; subcortical network; SZ, schizophrenia patients; HC healthy controls. *Between-group difference, FDR corrected *p* < 0.05.

**Figure 1.** Ten cortical functional networks described in Power et al. (2011) and 28 cerebellar regions from Diedrichsen (2006); and Diedrichsen, Balsters, Flavell, Cussans, and Ramnani (2009). Since the cortical networks consist of bilateral regions of interest, the hemispheric lobules in the cerebellum were merged in this study. *Abbreviations*. FPN, fronto-parietal network; CON, cingulo-opercular network; SAN, salience network; DAN; dorsal attention network; VAN, ventral attention network; DMN, default-mode network; MOT, motor network; AUD, auditory network; VIS, visual network; SUB, subcortical network.

**
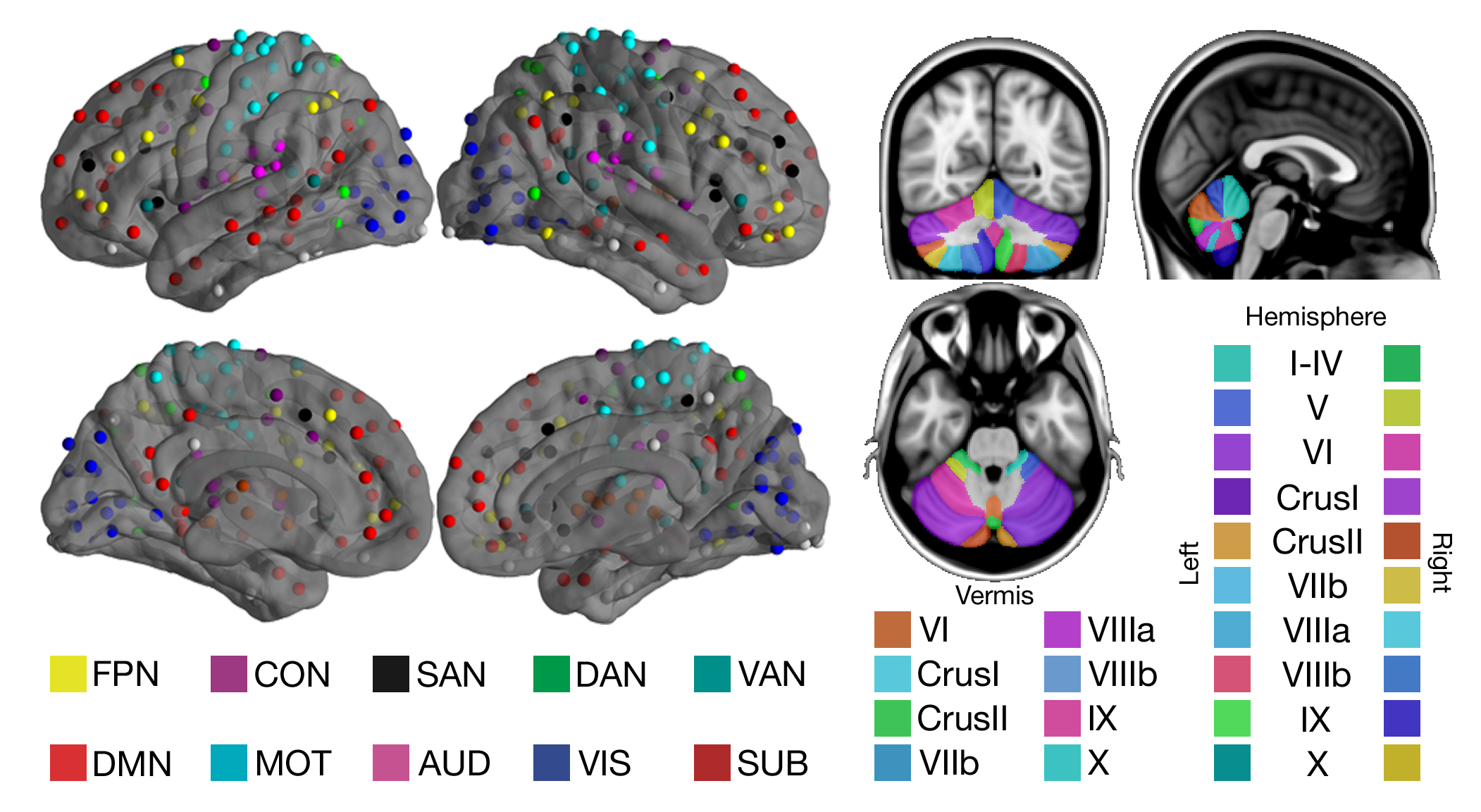
**

**Figure 2.** Group-averaged resting-state functional connectivity matrices with 227 cortical and 18 cerebellar regions, which identified putative functional networks. Abbreviations for the functional networks are the same as in Figure 1. *Abbreviations*. CB, cerebellum.


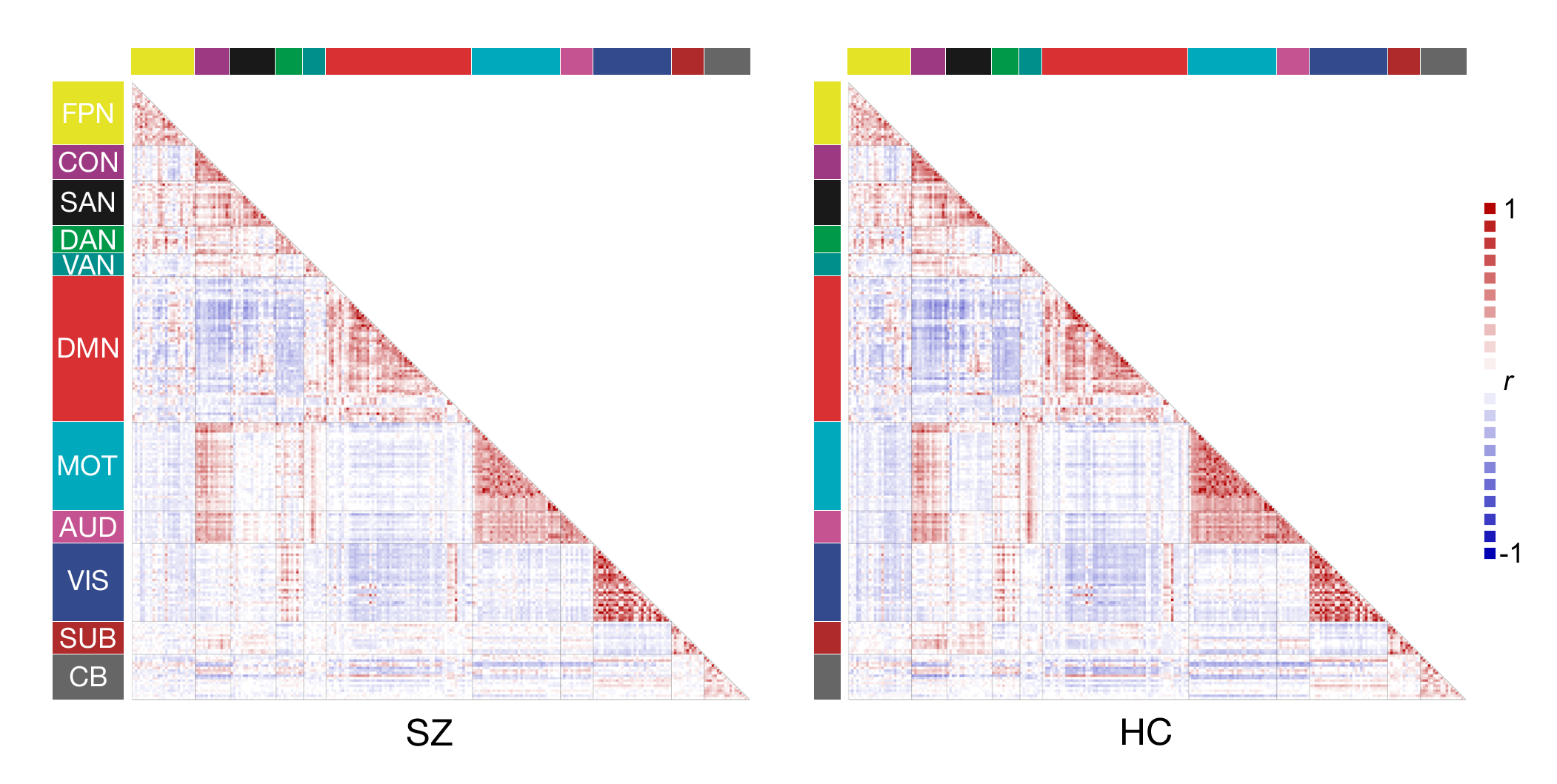


**Figure 3.** Correlations of cerebellar-cortical functional connectivity (FC) values between with and without global mean signal regression. Higher connectivity values with global signal regression also tend to have higher connectivity values even without global signal regression (i.e., positive correlations, *r* > 0.7, *p* < 0.001).

**
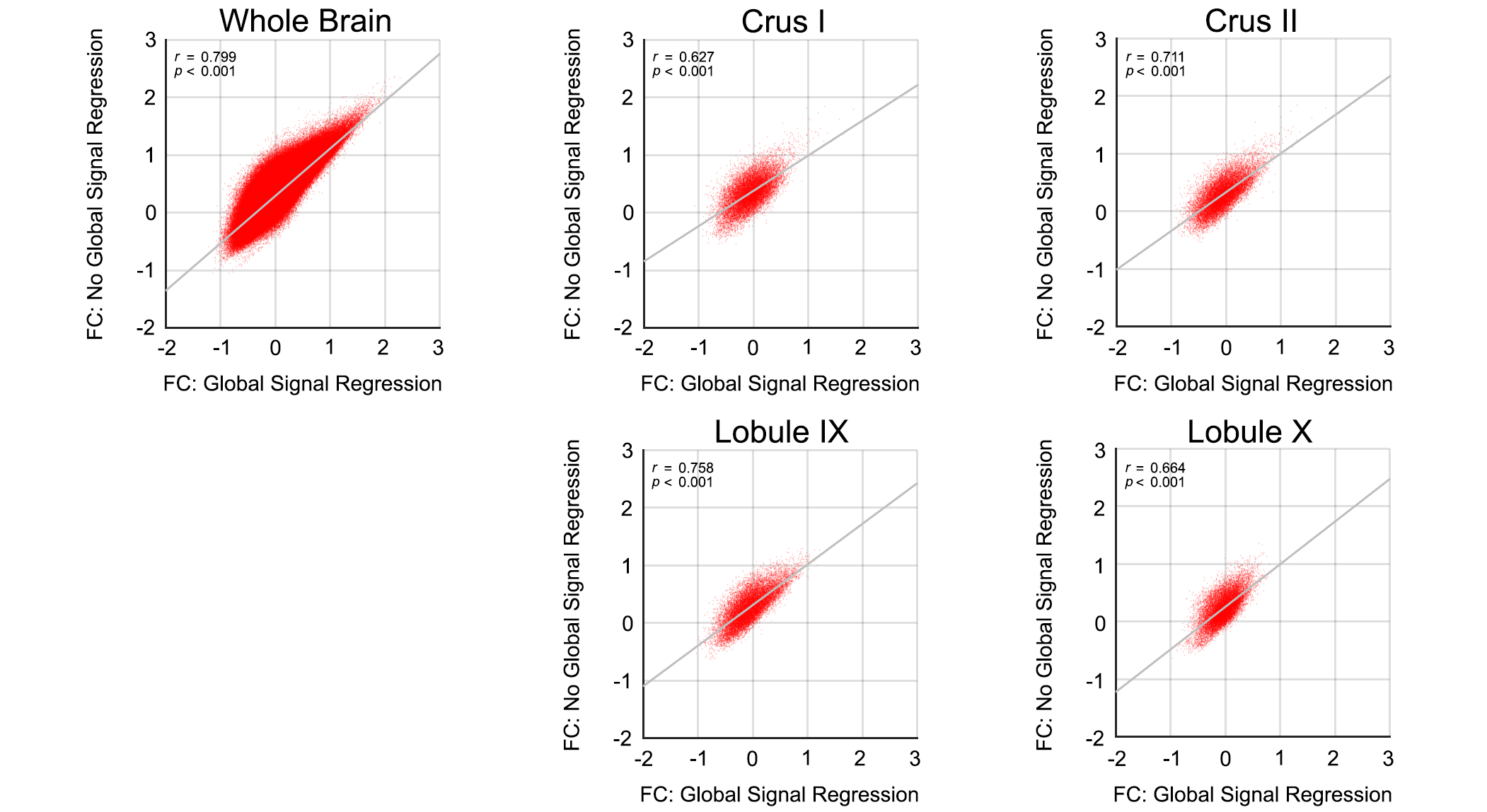
**

**Figure 4.** Comparison of cerebellar-cortical functional connectivity values between with and without global mean signal regression. Cerebellar-cortical functional connectivity with global signal regression (upper row) indicated between-group effects with uncorrected *p* < 0.05 (* marks). The functional connectivity without global signal regression (lower row) had no significant group differences (*p* > 0.05) while the patterns with higher functional connectivity in the schizophrenia patients were maintained.

**
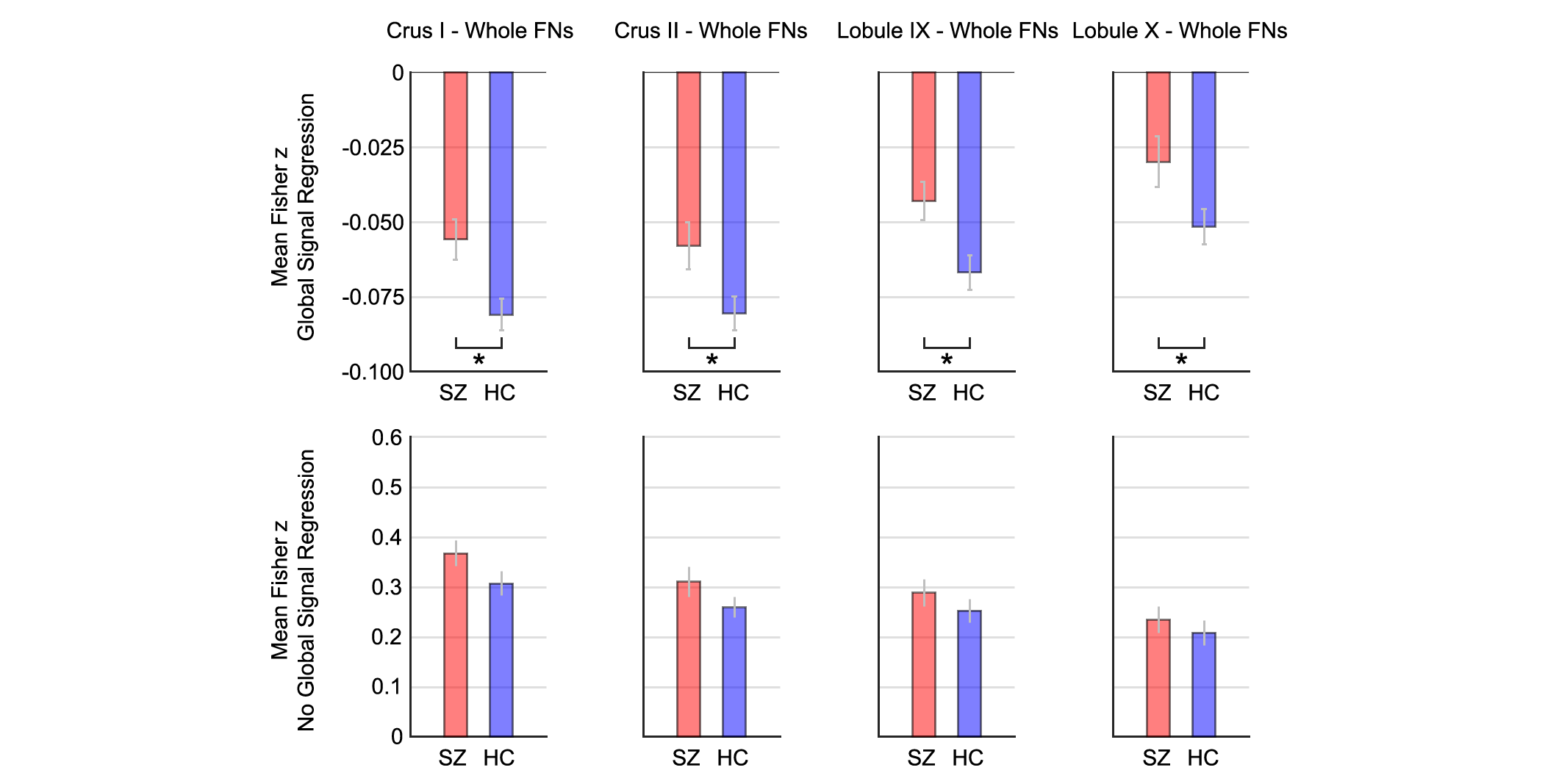
**

**Figure 5.** Comparison of the cerebellar-cortical functional connectivity without global mean signal regression between the cerebellum and cortical networks. No significant group differences were found (FDR-corrected *p* > 0.05) while the patterns with higher functional connectivity in the schizophrenia patients were comparable to those with global signal regression (See Figure 2 in the main text).

**
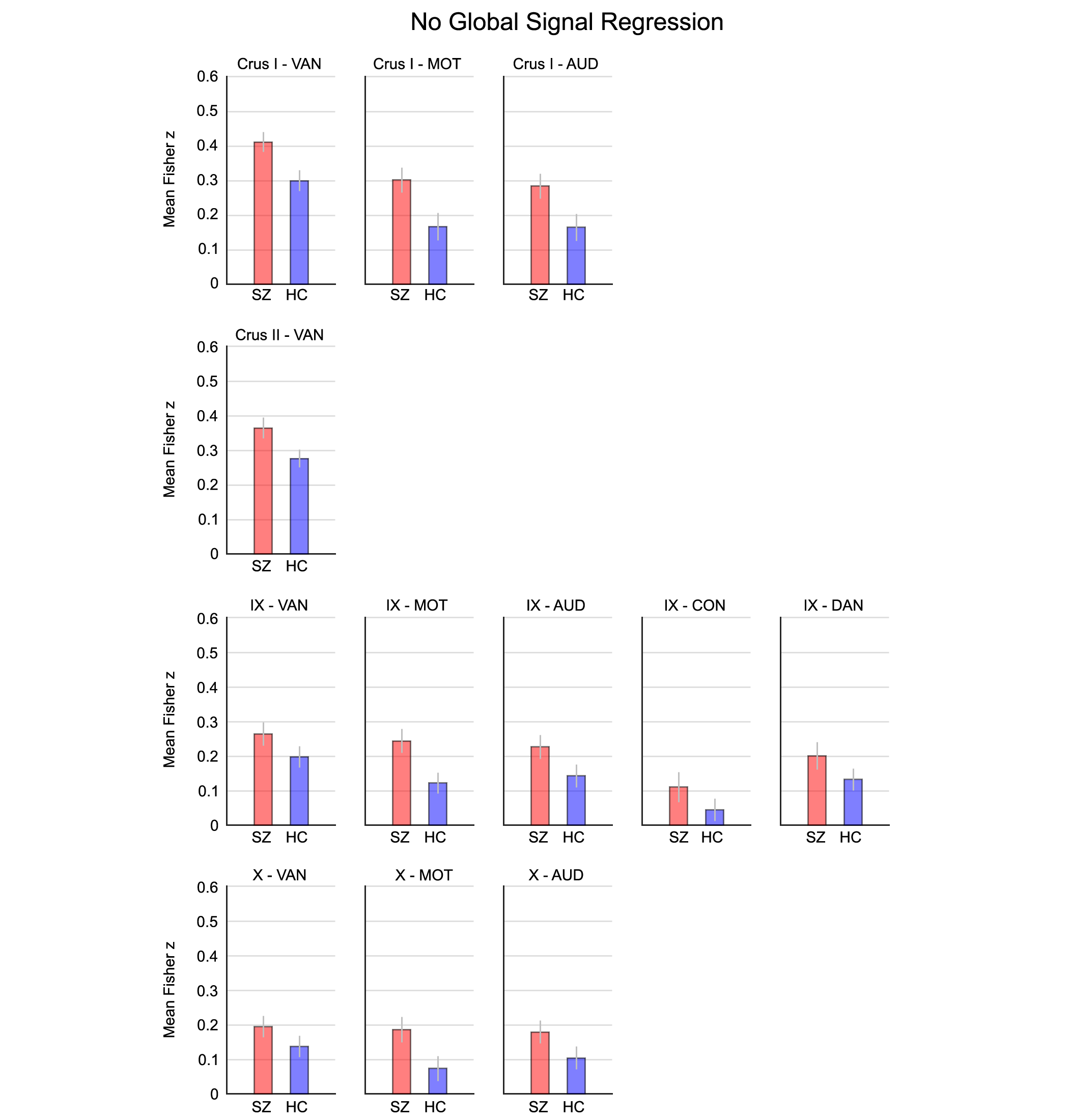
**

**Figure 6.** Cerebellar-cortical functional connectivity. Red and green labels represent between-group effects with FDR-corrected *p* < 0.05 and uncorrected *p* < 0.05, respectively. The scale of radar plots was ranged from -0.15 to 0.05 using increments of 0.05. Shaded areas and error bars represent ±1 SEM.


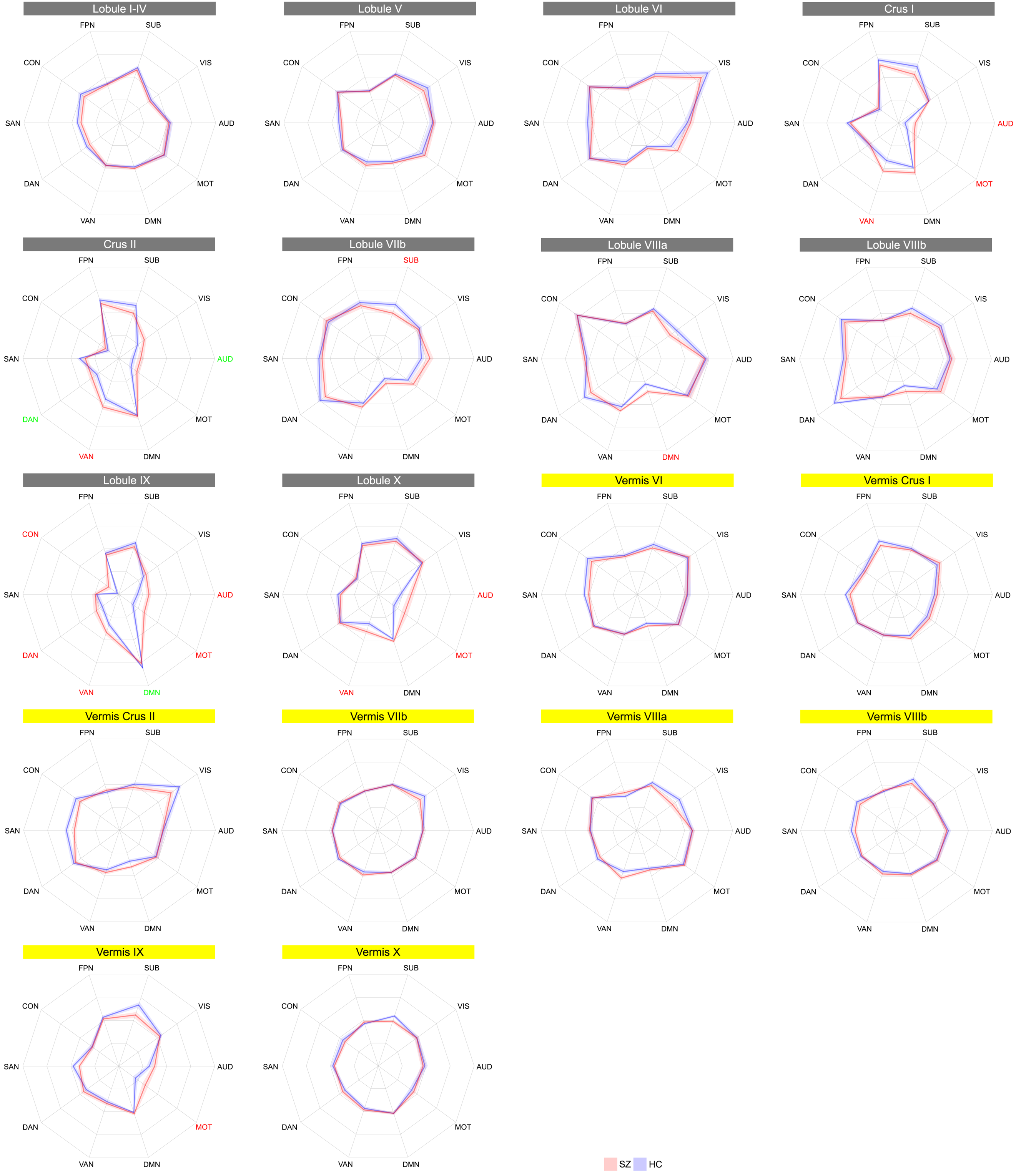


**Figure 7.** Behavioral analyses of the finger-tapping and postural sway measures. Statistical significance for between-group effects was defined at *p* < 0.05 (* marks).

**
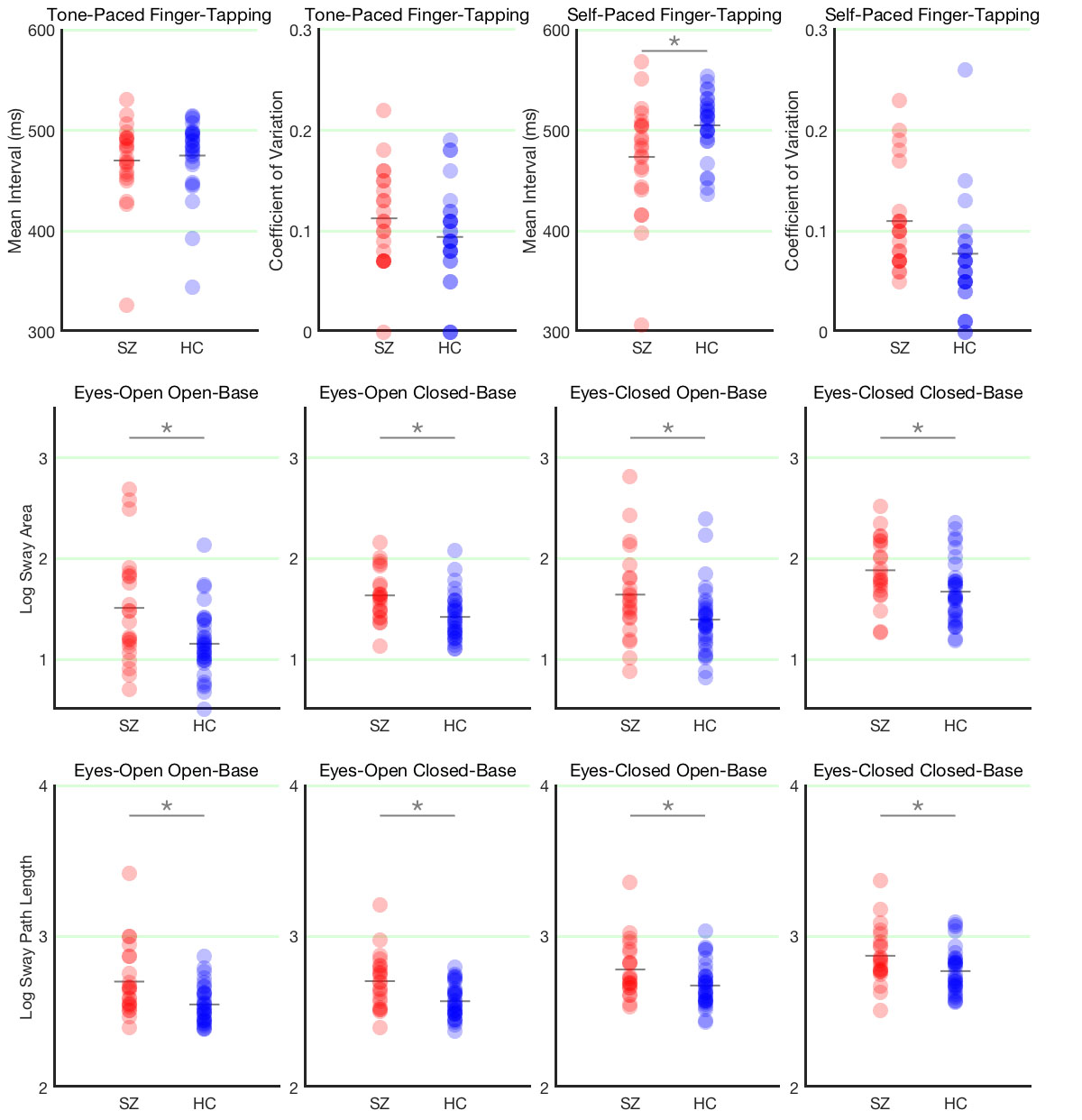
**

**Figure 8.** Significant associations between cerebellar-cortical functional connectivity (FC) and finger-tapping behavioral measures. Correlations were evaluated only in four altered lobules found in Figure 1-2, and significant associations in each group (*p* < 0.05) were indicated with gray solid lines. *Between-group effects between correlation coefficients of two groups at *p* < 0.05 (Fisher’s *z*-test).

**
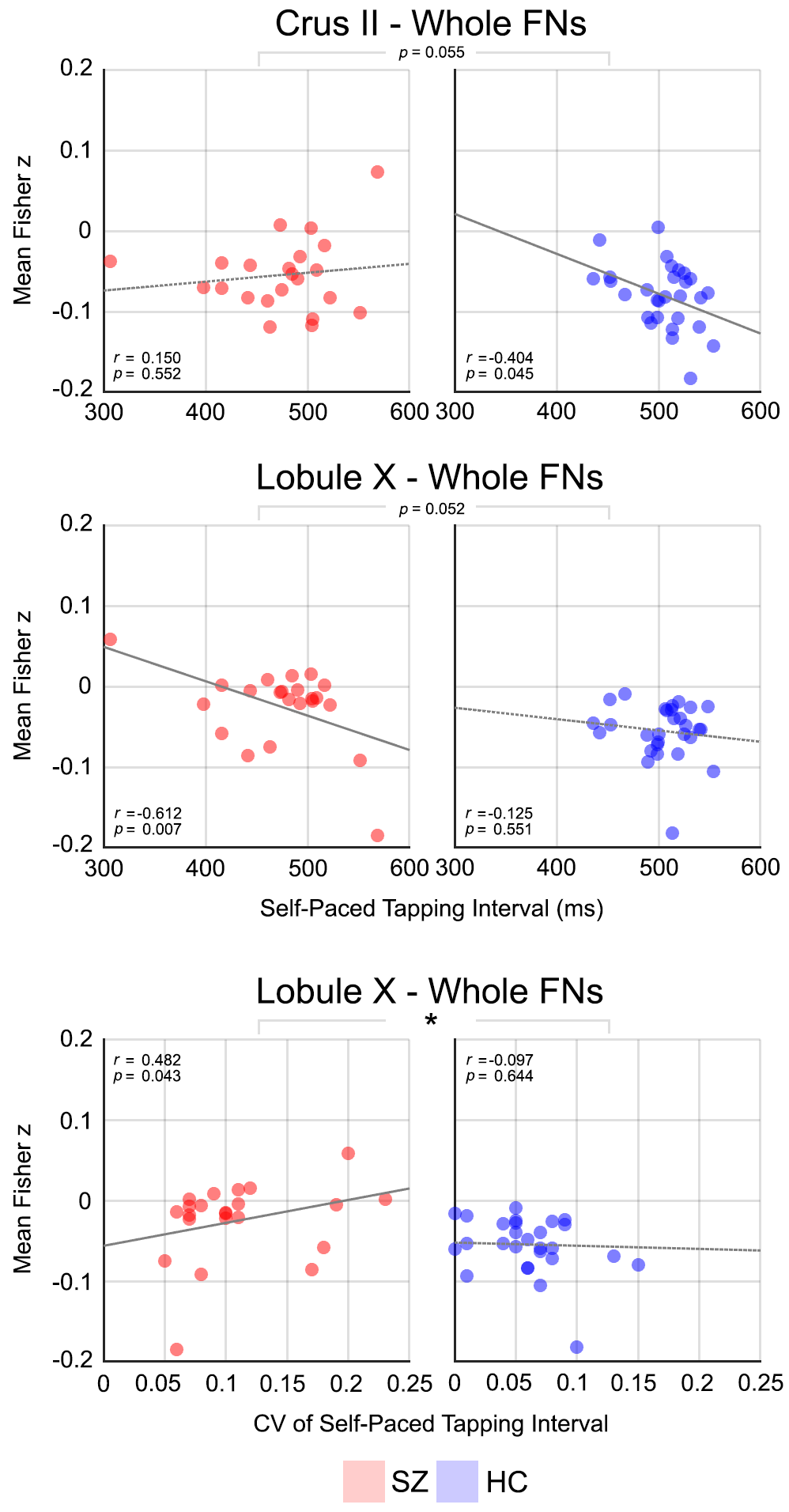
**

**References**

Diedrichsen, J. (2006). A spatially unbiased atlas template of the human cerebellum. *Neuroimage, 33*(1), 127-138. doi:10.1016/j.neuroimage.2006.05.056

Diedrichsen, J., Balsters, J. H., Flavell, J., Cussans, E., & Ramnani, N. (2009). A probabilistic MR atlas of the human cerebellum. *Neuroimage, 46*(1), 39-46. doi:10.1016/j.neuroimage.2009.01.045

Liu, T. T., Nalci, A., & Falahpour, M. (2017). The global signal in fMRI: Nuisance or Information? *Neuroimage, 150*, 213-229. doi:10.1016/j.neuroimage.2017.02.036

Murphy, K., Birn, R. M., Handwerker, D. A., Jones, T. B., & Bandettini, P. A. (2009). The impact of global signal regression on resting state correlations: are anti-correlated networks introduced? *Neuroimage, 44*(3), 893-905. doi:10.1016/j.neuroimage.2008.09.036

Murphy, K., & Fox, M. D. (2017). Towards a consensus regarding global signal regression for resting state functional connectivity MRI. *Neuroimage, 154*, 169-173. doi:10.1016/j.neuroimage.2016.11.052

Power, J. D., Cohen, A. L., Nelson, S. M., Wig, G. S., Barnes, K. A., Church, J. A., . . . Petersen, S. E. (2011). Functional network organization of the human brain. *Neuron, 72*(4), 665-678. doi:10.1016/j.neuron.2011.09.006

Power, J. D., Plitt, M., Gotts, S. J., Kundu, P., Voon, V., Bandettini, P. A., & Martin, A. (2018). Ridding fMRI data of motion-related influences: Removal of signals with distinct spatial and physical bases in multiecho data. *Proc Natl Acad Sci U S A, 115*(9), E2105-E2114. doi:10.1073/pnas.1720985115

Yang, G. J., Murray, J. D., Glasser, M., Pearlson, G. D., Krystal, J. H., Schleifer, C., . . . Anticevic, A. (2017). Altered Global Signal Topography in Schizophrenia. *Cereb Cortex, 27*(11), 5156-5169. doi:10.1093/cercor/bhw297

Yang, G. J., Murray, J. D., Repovs, G., Cole, M. W., Savic, A., Glasser, M. F., . . . Anticevic, A. (2014). Altered global brain signal in schizophrenia. *Proc Natl Acad Sci U S A, 111*(20), 7438-7443. doi:10.1073/pnas.1405289111
